# Fibroblast Growth Factor Type 1 Ameliorates High-Glucose-Induced Oxidative Stress and Neuroinflammation in Retinal Pigment Epithelial Cells and a Streptozotocin-Induced Diabetic Rat Model

**DOI:** 10.3390/ijms22137233

**Published:** 2021-07-05

**Authors:** Hsin-Wei Huang, Chung-May Yang, Chang-Hao Yang

**Affiliations:** 1 Department of Ophthalmology, Wan Fang Hospital, Taipei Medical University, No. 111, Sec. 3, Xinglong Rd., Taipei 11696, Taiwan; 100320@w.tmu.edu.tw; 2 Graduate Institute of Clinical Medicine, College of Medicine, National Taiwan University, No. 1, Jen Ai Road Sec. 1, Taipei 100, Taiwan; 3 Department of Ophthalmology, National Taiwan University Hospital, No. 7, Zhongshan South Road, Taipei 100, Taiwan; chungmay@ntu.edu.tw; 4 Department of Ophthalmology, College of Medicine, National Taiwan University, No. 1, Jen Ai Road, Sec. 1, Taipei 100, Taiwan

**Keywords:** fibroblast growth factor type 1, diabetic retinopathy, oxidative stress, inflammatory mediator, p38 mitogen-activated kinase, nuclear factor-κB

## Abstract

Diabetic retinopathy (DR) is a common complication of diabetes that causes severe visual impairment globally. The pathogenesis of DR is related to oxidative stress and chronic inflammation. The fibroblast growth factor type 1 (FGF-1) mitogen plays crucial roles in cell function, development, and metabolism. FGF-1 is involved in blood sugar regulation and exerts beneficial antioxidative and anti-inflammatory effects on various organ systems. This study investigated the antioxidative and anti-inflammatory neuroprotective effects of FGF-1 on high-glucose-induced retinal damage. The results revealed that FGF-1 treatment significantly reversed the harmful effects of oxidative stress and inflammatory mediators in retinal tissue in a streptozotocin-induced diabetic rat model. These protective effects were also observed in the in vitro model of retinal ARPE-19 cells exposed to a high-glucose condition. We demonstrated that FGF-1 attenuated p38 mitogen-activated protein kinase and nuclear factor-κB pathway activation under the high-glucose condition. Our results indicated that FGF-1 could effectively prevent retinal injury in diabetes. The findings of this study could be used to develop novel treatments for DR that aim to reduce the cascade of oxidative stress and inflammatory signals in neuroretinal tissue.

## 1. Introduction

Diabetic retinopathy (DR) is a common complication of diabetes mellitus and the main cause of visual impairment globally [1,2,3,4]. Numerous methods have been used to reduce visual impairment caused by DR including retinal laser therapy [5], intravitreal injection of antiangiogenic drugs [6,7], intravitreal injection of steroids [8], and vitrectomy [9,10,11]. However, DR progression has not been effectively and completely prevented [12]. Therefore, a molecular target that can be used as a new treatment strategy should be identified.

The pathogenesis of DR is complex and not yet fully understood. The relationship between oxidative stress and diabetic complications has been known for over 20 years [13]. Oxidative stress refers to a severe imbalance between oxidants (reactive nitrogen species and reactive oxygen species [ROS]) and antioxidants [14]. The formation and accumulation of advanced glycation end products and advanced lipoxidation end products are pathways for the initiation and progression of DR [15,16,17]. Oxidatively modified DNA, proteins, and lipids (i.e., 8-hydroxydeoxyguanosine [8-OHdG], nitrotyrosine, and acrolein) are sensitive biomarkers of increased oxidative stress [18]. Increased expression of these biomarkers was detected in the retina of diabetic rats [19]. 8-OHdG staining was localized in the inner segments of the photoreceptor, inner nuclear, outer plexiform, and inner plexiform layers; such staining was markedly decreased in the inner segments and outer plexiform layer [20]. Nitrotyrosine immunoreactivity was strongly localized in photoreceptor inner segments, outer and inner plexiform layers [21]. Acrolein was localized in the Müller glial cells of diabetic rats and the human retina [22].

Inflammation is highly correlated with DR [23,24]. ROS are considered strong inflammatory factors that can stimulate the synthesis of many cytokines and chemokines including intercellular adhesion molecule 1 (ICAM-1), monocyte chemoattractant protein-1 (MCP-1), interleukin (IL)-1β, and IL-6. These cytokines and chemokines participate in the development of diabetic retinal disease [25]. Therefore, effective control of oxidative stress and inflammation can provide therapeutic benefits for diabetes-related retinal neurodegeneration.

Fibroblast growth factor type 1 (FGF-1, also referred to as acidic fibroblast growth factor) is a 155-amino acid polypeptide that is released from cells through a nonclassical secretory pathway [26]. FGF-1 was first isolated as a mitogen for fibroblasts in the bovine brain and pituitary glands [27]. FGF-1 regulates the mitotic processes of various tissues and cells such as systemic vascular cells and skin cells [28,29,30]. Clinically, human FGF-1 can be used to promote the repair process of burn wounds and the regeneration of ulcer wounds [31]. Furthermore, FGF-1 exerts cardioprotective effects against myocardial disease caused by ischemia-reperfusion injury [32,33,34]. In addition, FGF-1 can be used to treat human cervical spinal cord injuries [35].

In diabetes-related diseases, FGF-1 regulates blood glucose levels and insulin resistance without causing hypoglycemia in diabetic mice [36]. Furthermore, FGF-1 may be used to treat diabetes-related complications (i.e., those related to the heart, liver, kidney, and skin). FGF-1 could prevent diabetic cardiomyopathy in rats, mice, and H9C2 cardiomyocytes by attenuating high-glucose-induced cardiac hypertrophy, oxidative stress, and myocardial fibrosis [37,38]. In addition, FGF-1 effectively reduced liver damage in a mouse model of fatty liver disease [39] and reduced renal inflammation, glomeruli, and renal tubule damage, and renal dysfunction in a diabetic mouse model [40]. Moreover, FGF-1 has been used to treat refractory wounds in diabetic rats because of its mitogenic activity [41]. The FGF-1 localization pattern has been reported in the rat [42], bovine [43], and human [44] retinas. FGF-1 was found to be localized in ganglion cells, horizontal cells, photoreceptors in their inner and outer segments, and retinal pigment epithelial (RPE) cells [45,46,47]. In primary cell culture, RPE cells could produce FGF-1 [48]. However, the neuroprotective effects of FGF-1 on diabetic retinal diseases have not been extensively studied.

In the present study, we used streptozotocin (STZ)-induced diabetic rats as an in vivo model and high-glucose-induced human ARPE-19 cells as an in vitro model to investigate the antioxidative and anti-inflammatory neuroprotective effects of FGF-1 on high-glucose-induced retinal damage.

## 2. Results

### 2.1. Suppression of Endogenous FGF-1 Levels in STZ-Induced Diabetic Rats

**Figure 1:**
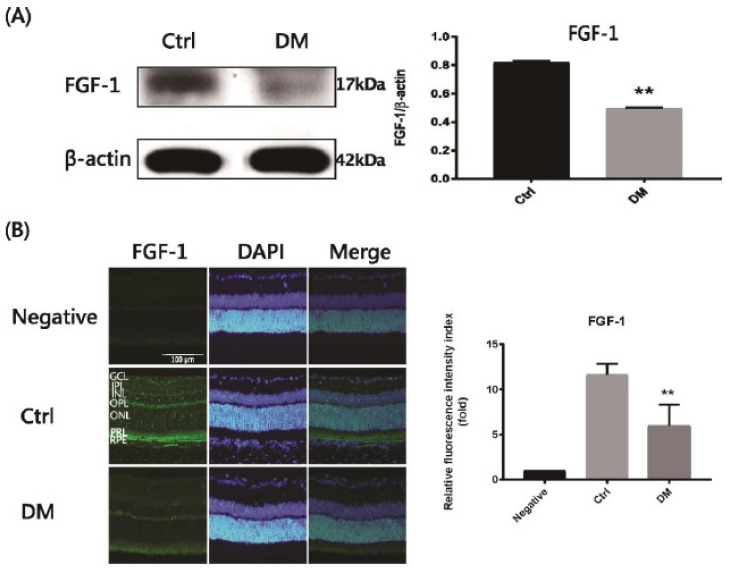
Endogenous levels of FGF-1 in diabetic Sprague-Dawley (SD) rats. (**A**) Western blot of FGF-1 in the retinal tissues of 12-week-old SD rats. The bar graph illustrates the relative intensity of western blot bands, normalized to β-actin expression (*n* = 4 eyes in each group). (**B**) Immunofluorescence image of FGF-1 expression in the retinal tissue of 12-week-old SD rats. Bar = 100 μm. The relative densities of immunofluorescence images were normalized to the negative control (*n* = 4 eyes in each group). All data are presented as mean ± standard deviation. ** *p* < 0.01 compared with the control group; Ctrl, control group; DAPI, 4′,6-diamidino-2-phenylindole; FGF-1, fibroblast growth factor type 1; DM, STZ- induced diabetic rat group; Negative, negative control group.

First, we investigated the FGF-1 protein level in STZ-induced diabetic rats and nondiabetic control rats through western blotting (WB) and immunofluorescence (IF). WB results indicated that the FGF-1 protein level was significantly lower in diabetic rat retinas than in the control rat retinas (Figure 1A). Furthermore, IF results showed that the FGF-1 protein level was significantly lower in diabetic rat retinas than in the control rat retinas (Figure 1B). Differences between the groups observed in WB and IF analyses were statistically significant. We found that the endogenous FGF-1 level was markedly suppressed in diabetic rats. Thus, we investigated the effect of FGF-1 treatment on the restoration of the FGF-1 level in DR.

### 2.2. FGF-1 Treatment Prevents DR in STZ-Induced Diabetic Rats by Reducing Oxidative Stress

**Figure 2:**
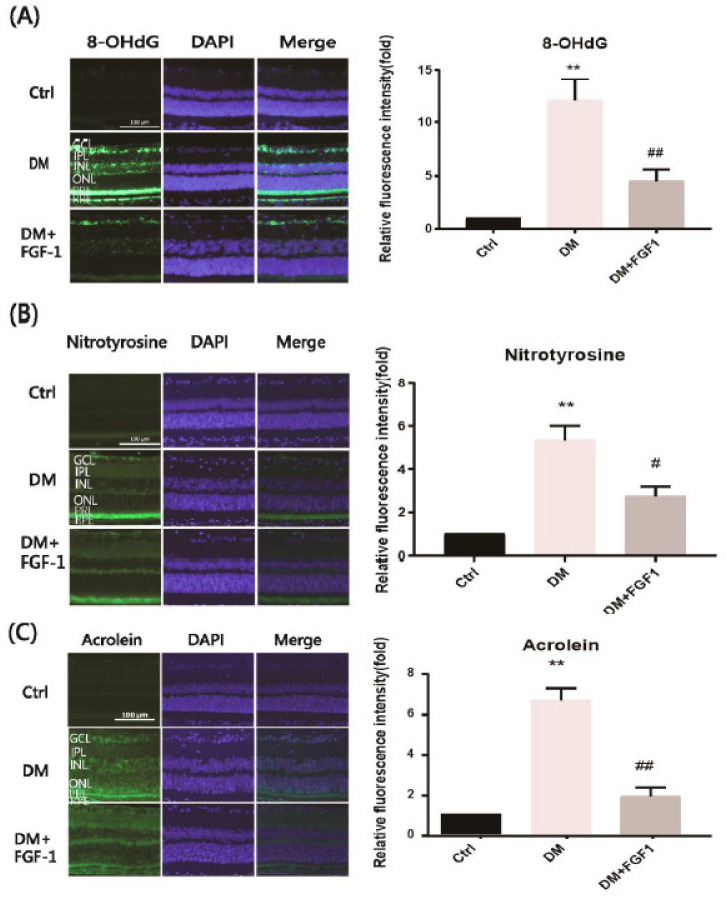
FGF-1 treatment reduced oxidative stress in the rat retinas. Retinal sections were collected from 12-week-old SD rats in each group. Immunofluorescence imaging of the expression of (**A**) 8-OHdG, (**B**) nitrotyrosine, and (**C**) acrolein in the retinal tissue (*n* = 4 eyes in each group). Bar = 100 µm. The relative densities of immunofluorescence were quantified using ImageJ software. Data are presented as mean ± standard deviation; ** *p* < 0.01 versus the control group; #*p* < 0.05, ## *p* < 0.01 versus the DM group. DAPI, 4′,6-diamidino-2-phenylindole; Ctrl, control group; DM, STZ-induced diabetic rat group; DM + FGF-1, fibroblast growth factor type 1 treatment group.

To investigate the potential effect of FGF-1 treatment in diabetes, intravitreal injections of FGF-1 (1.5 μg/2 μL) once per week for one month were administered to treat STZ-induced diabetic rats. We previously identified 8-OHdG, nitrotyrosine, and acrolein as oxidative stress indicators generated by DNA, protein, and lipid oxidation, respectively. Therefore, we examined these indicators in the rat retina to evaluate oxidative stress conditions in STZ-induced diabetic rats. We performed an IF analysis of the rat retinas to detect the three oxidative stress-associated indicators (Figure 2). The IF staining of each indicator molecule was quantified using ImageJ software. The results are presented graphically. In the control group, the IF of these three oxidative stress indicators was considerably low. IF was strong in the STZ-induced DM group. Subsequently, we used FGF-1 to examine the effect of FGF-1 treatment on oxidative stress in DR. The FGF-1 treatment group exhibited a significant reduction in these three indicators compared with the DM group (*p* < 0.05). FGF-1 treatment effectively reversed diabetic changes in these three oxidative stress indicators. The findings indicate that FGF-1 treatment reduced the diabetes-induced expression of 8-OHdG, nitrotyrosine, and acrolein in rat retinas by reducing oxidative stress.

### 2.3. FGF-1 Treatment Attenuates Diabetes-Induced Inflammatory Mediators in the Retinas of STZ-Induced Diabetic Rats

**Figure 3:**
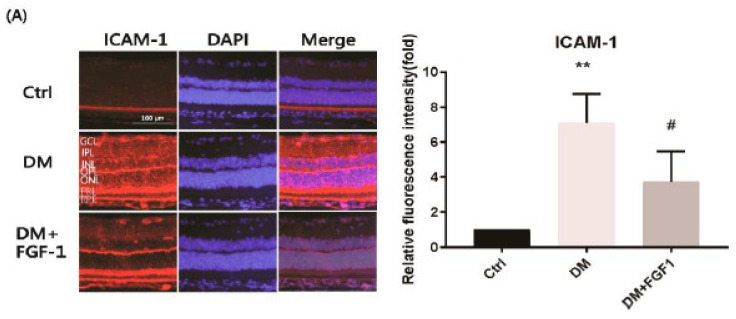

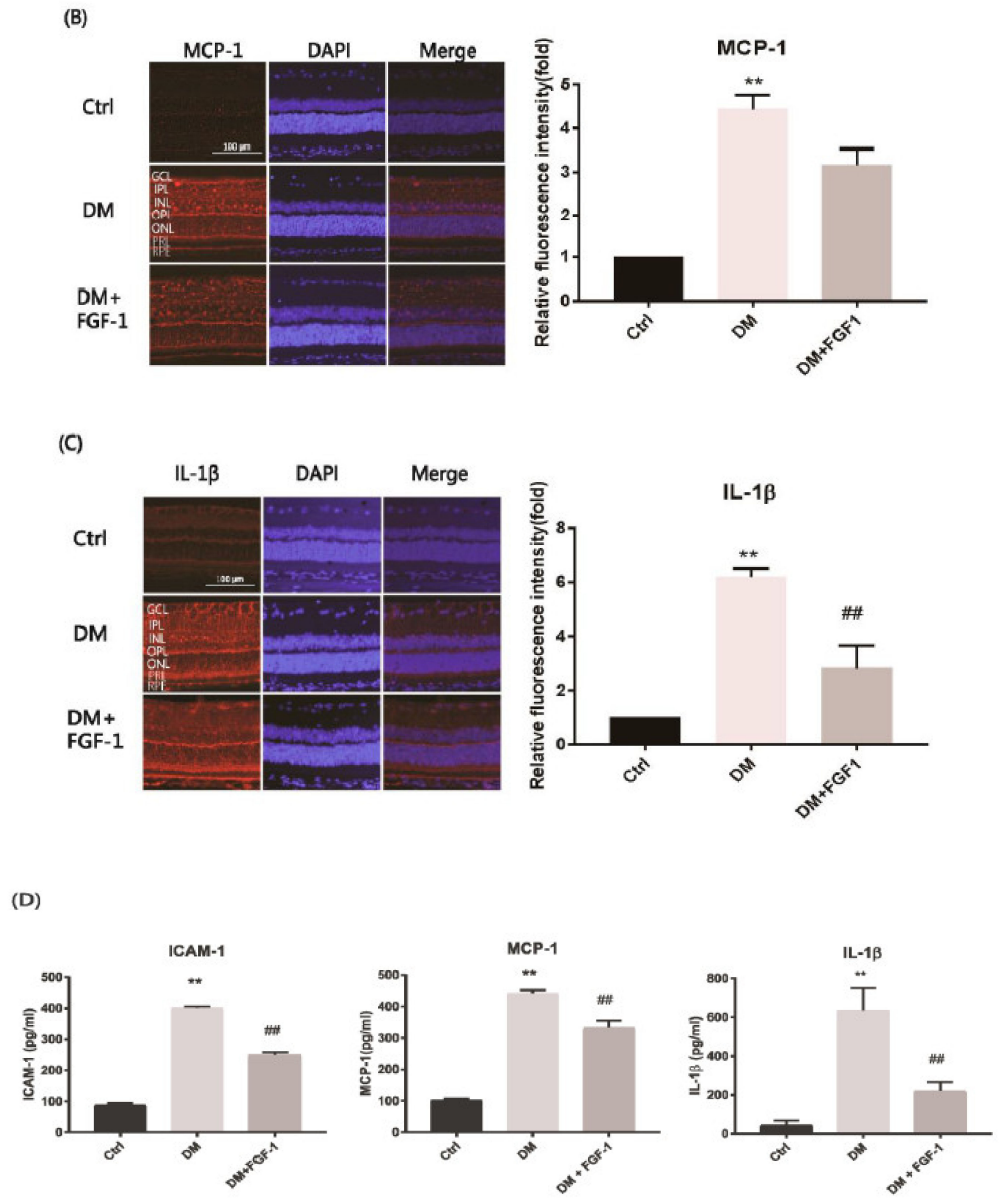
FGF-1 treatment attenuated inflammatory mediators in rats. Retinal sections were collected from 12-week-old SD rats in each group. Immunofluorescence images of inflammatory mediators (**A**) ICAM-1, (**B**) MCP-1, and (**C**) IL-1β in retinal tissue. The relative densities of immunofluorescence images were quantified using ImageJ software and are presented in bar graphs (*n* = 4 eyes in each group). (**D**) Aqueous humor was collected from the rat eyeballs. ICAM-1, MCP-1, and IL-1β protein levels were measured in each group using ELISA. Each assay was repeated three times (*n* = 4 eyes in each group). All data are presented as mean ± standard deviation; ** *p* < 0.01 compared with the control group; # *p* < 0.05, ## *p* < 0.01 compared with the diabetes group. DAPI, 4′,6-diamidino-2-phenylindole; Ctrl, control group; DM, STZ-induced diabetic rat group; DM + FGF-1, fibroblast growth factor type 1 treatment group.

Because inflammation is a main cause of DR, the expression of inflammatory mediators can be used as an index of diabetic retinal injury. In DR, ICAM-1, MCP-1, and IL-1β are considered as potent inflammatory cytokines. ICAM-1 was localized in the ganglion layer, external limiting membrane, RPE/Bruch’s membrane, retinal blood vessels, and choroidal vessels [49]. MCP-1 was predominantly expressed in the retinal ganglion cell layer and inner nuclear layer [50]. In addition, MCP-1 was localized in the outer plexiform layer and Müller cells [51]. IL-1β localization was primarily observed in the outer retina and subretinal space [52]. Furthermore, IL-1β was found to be localized in unspecified cells in the inner retina in the Akimba mouse model of PDR [53]. In other studies, IL-1β has been observed to be localized in Müller cells and RGCs [54]. Therefore, we evaluated the expression levels of these inflammatory cytokines through IF staining in the whole retinas that were untreated or treated with FGF-1. The treatment effects of FGF-1 on ICAM-1, MCP-1, and IL-1β expression levels are presented in Figure 3A–C, respectively. The expression of the three inflammatory proteins significantly increased in the retinas of the diabetic group. IF was weaker in the FGF-1 treatment group, indicating lower cytokine expression.

ImageJ software was used to quantify relative IF staining intensities. The bar graphs in Figure 3A–C indicate that the values of these inflammatory proteins in the diabetic retinas were significantly higher than those in the control group (*p* < 0.05). The expression levels of ICAM-1 and IL-1β were lower in the FGF-1 treatment group than in the diabetes group (*p* < 0.05). Although MCP-1 expression in the FGF-1 treatment group did not significantly differ from that in the diabetic group, MCP-1 expression was generally lower in the FGF-1 treatment group (Figure 3B). These results strongly suggest that FGF-1 has a protective role against diabetes-induced inflammation in the rat retina.

We measured the concentrations of the inflammatory cytokines ICAM-1, MCP-1, and IL-1β in the rat aqueous humor (AqH) that was untreated or treated with FGF-1 by using the enzyme-linked immunosorbent assay (ELISA). FGF-1 treatment significantly reduced ICAM-1, MCP-1, and IL-1β levels in the AqH (*p* < 0.01; Figure 3D). The data suggest that FGF-1 treatment could ameliorate the levels of diabetes-induced inflammatory mediators in the retinas of diabetic rats.

### 2.4. FGF-1 Treatment Ameliorates Oxidative Stress Damage in ARPE-19 Cells Exposed to Elevated Glucose Levels

**Figure 4:**
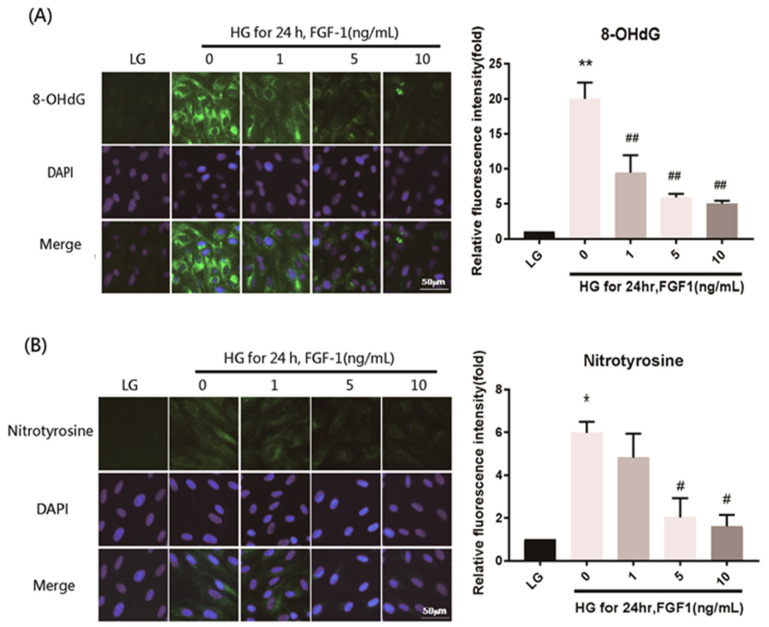

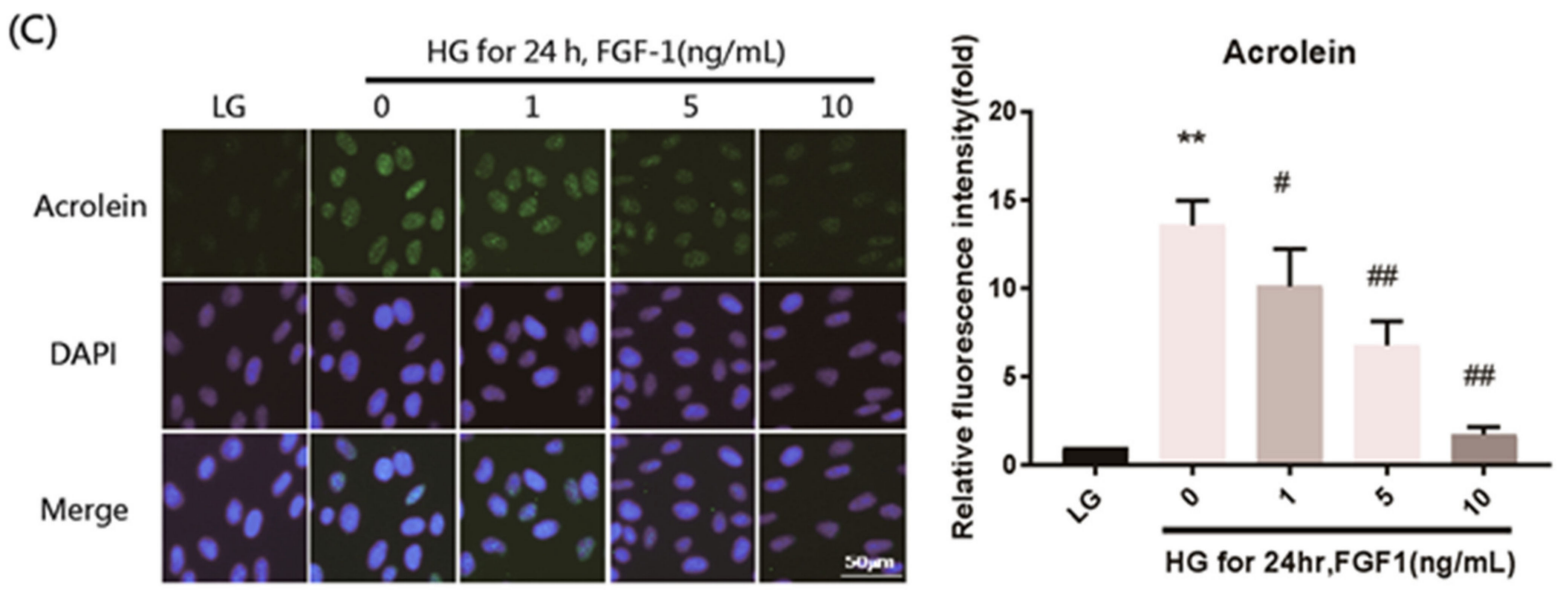
FGF-1 treatment ameliorated cell oxidative stress in ARPE-19 cells incubated in high-glucose medium. After pretreatment with a series of different concentrations of FGF-1 (1, 5, and 10 ng/mL) for 2 h, ARPE-19 cells were incubated in a high-glucose (25 mM) medium for 24 h. Expression of oxidative stress mediators (**A**) 8-OHdG, (**B**) nitrotyrosine, and (**C**) acrolein were detected using immunofluorescence (IF) imaging (*n* = 5 in each group). The relative fluorescence signals of the IF image were quantified using ImageJ software. * *p* < 0.05, ** *p* < 0.01 compared with the control cells incubated in the low-glucose medium; # *p* < 0.05, ## *p* < 0.01 compared with ARPE-19 cells incubated in high-glucose medium. DAPI, 4′,6-diamidino-2- phenylindole; LG, low glucose; HG, high glucose; FGF-1, fibroblast growth factor type 1.

Hyperglycemia leads to several biochemical consequences including oxidative stress, which causes retinal injury. We used ARPE-19 cells to examine the mechanism of FGF-1 treatment. We performed IF imaging to examine the performance of the peroxidation products 8-OHdG, nitrotyrosine, and acrolein in ARPE-19 cells and determine the mechanism of FGF-1 treatment in oxidative stress damage induced by a high-glucose medium. Oxidative stress increased after the incubation of the cells in the high-glucose medium, as evidenced by the increased expression (increased IF) of the three oxidative stress indicators. These changes caused by high-glucose stimulation were reversed by FGF-1 treatment. The downward trend was related to FGF-1 concentration (1, 5, and 10 ng/mL FGF-1; Figure 4). These results indicate that FGF-1 treatment could prevent high glucose level–induced oxidative stress in ARPE-19 cells.

### 2.5. FGF-1 Treatment Alleviates High-Glucose-Stimulated Inflammatory Mediators in ARPE-19 Retinal Cells

**Figure 5:**
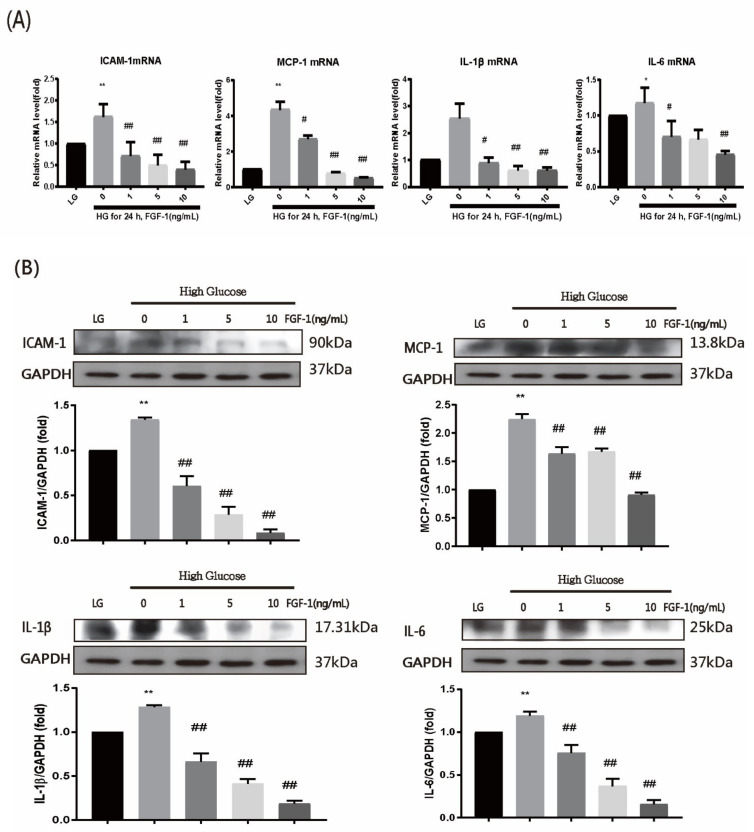

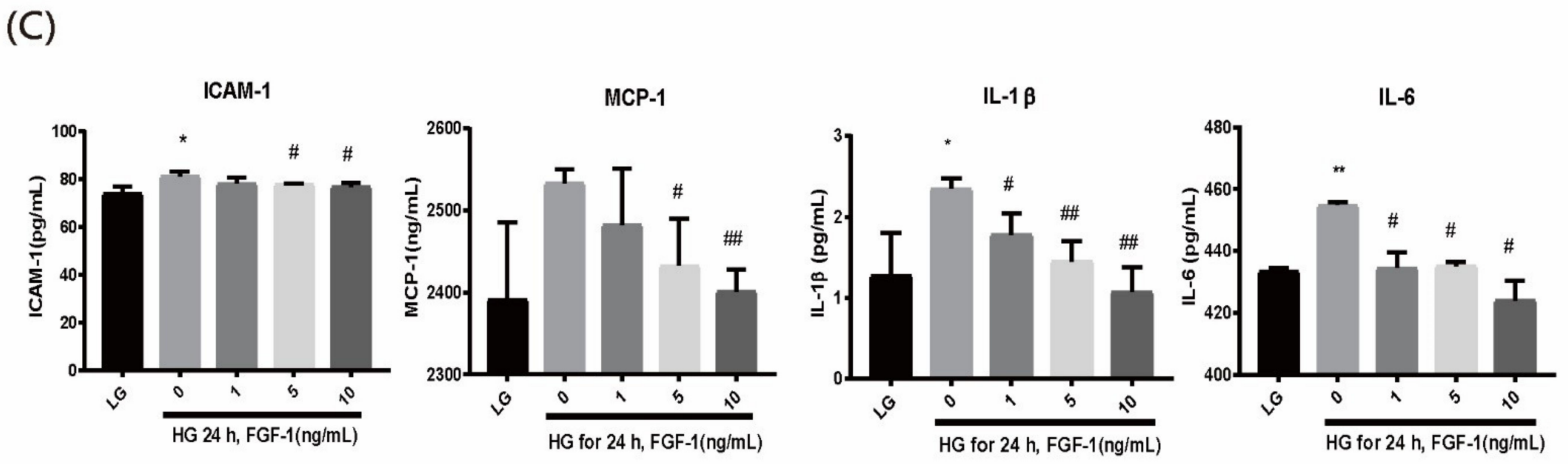
FGF-1 treatment alleviates cell inflammation in ARPE-19 cells incubated in high-glucose medium. After pretreatment with different concentrations of FGF-1 (1, 5, and 10 ng/mL) for 2 h, ARPE-19 cells were incubated in a high-glucose (25 mM) medium for 24 h. (**A**) Expression levels of inflammatory mediators (ICAM-1, MCP-1, IL-1β, and IL-6) were detected using RT-PCR. (**B**) Protein expression of inflammatory mediators was assessed using western blot analysis. (**C**) Protein expression of inflammatory mediators in cell culture medium was assessed using ELISA (*n* = 5 in each group). * *p* < 0.05, ** *p* < 0.01 compared with control cells incubated in low-glucose medium; # *p* < 0.05, ## *p* < 0.01 compared with ARPE-19 cells incubated in high-glucose medium. LG, low glucose; HG, high glucose; FGF-1, fibroblast growth factor type 1; GAPDH, glyceraldehyde-3-phosphate dehydrogenase.

To examine whether ARPE-19 cells incubated in the high-glucose medium exhibited the same inflammatory response as diabetic rats, we measured the mRNA levels of ICAM-1, MCP-1, IL-1β, and IL-6 through quantitative reverse transcription-polymerase chain reaction (RT-PCR; Figure 5A). All four mRNA levels were significantly higher in the high-glucose group than in the low-glucose and FGF-1 treatment groups. The downward trend was related to the concentration of FGF-1 treatment (1, 5, and 10 ng/mL FGF-1). A higher FGF-1 concentration was associated with better protection than that conferred by a lower FGF-1 concentration.

WB analysis was performed to confirm the expression of the inflammatory mediators ICAM-1, MCP-1, IL-1β, and IL-6 in ARPE-19 cells. The levels of all four inflammatory mediators were increased in the high-glucose medium (Figure 5B). FGF-1 treatment reduced the levels of the four inflammatory substances. A higher FGF-1 concentration was more effective than a lower FGF-1 concentration in protecting against diabetes-induced inflammation.

The concentrations of ICAM-1, MCP-1, IL-6, and IL-1β in the ARPE-19 cell culture medium were measured using ELISA. Groups treated with FGF-1 exhibited significantly decreased ICAM-1, MCP-1, IL-6, and IL-1β levels (*p* < 0.05; Figure 5C). Additionally, a higher FGF-1 concentration was more effective than a lower FGF-1 concentration was in protecting against high-glucose-induced inflammation. These results indicate that FGF-1 could prevent high-glucose-induced inflammation in ARPE-19 cells.

### 2.6. FGF-1 Suppresses the Activation of P38 and NF-κB Signaling in ARPE-19 Cells Incubated in High-Glucose Medium

**Figure 6:**
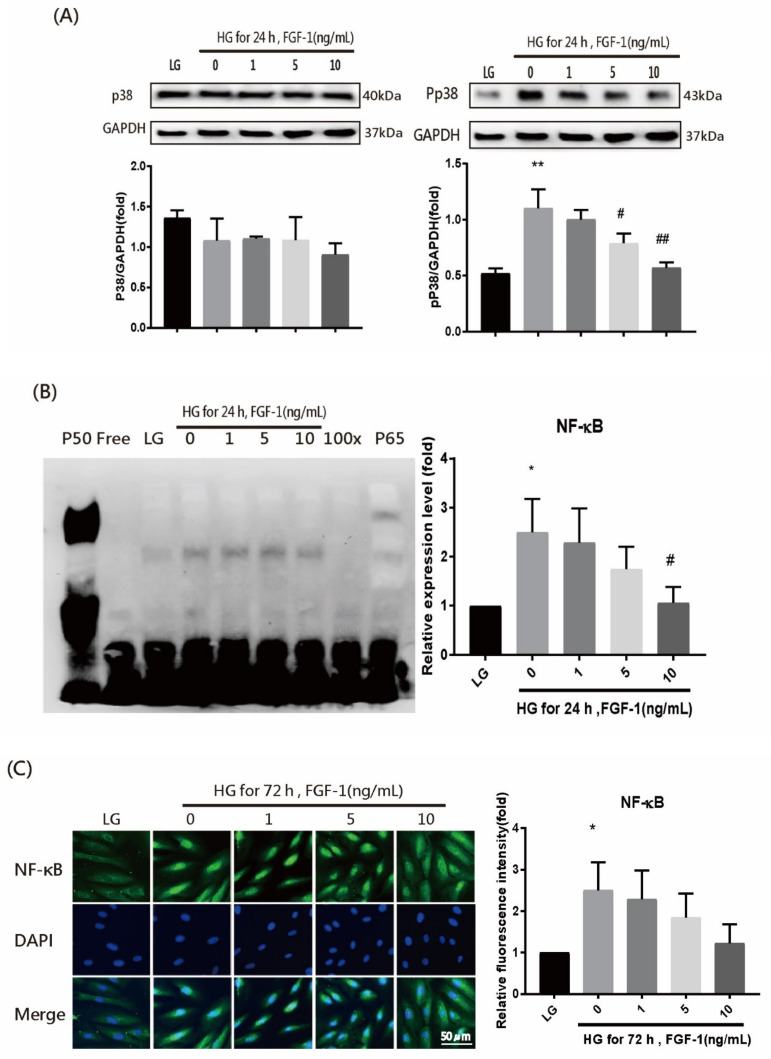
FGF-1 suppresses p38 and NF-κB signaling in ARPE-19 cells incubated in high-glucose medium. After pretreatment with different concentrations of FGF-1 (0, 1, 5, and 10 ng/mL) for 2 h, ARPE-19 cells were incubated in high-glucose (25 mM) medium for 24 or 72 h. (**A**) Expression levels of p38 and Pp38 proteins were assessed through WB. The relative intensities of the bands were normalized to the expression of glyceraldehyde 3-phosphate dehydrogenase (GAPDH; *n* = 5 in each group). (**B**) EMSA was performed by incubating nuclear extracts with the NF-κB consensus sequence. Lane 1—p50 subunit of NF-κB; lane 2—free probe; lane 3—low-glucose ARPE-19; lane 4—high-glucose ARPE-19; lane 5—high-glucose ARPE-19 with 1 ng/mL FGF-1; lane 6—high-glucose ARPE-19 with 5 ng/mL FGF-1; lane 7—high-glucose ARPE-19 with 10 ng/mL FGF-1; lane 8—100-fold molar excess of unlabeled NF-κB probe; and lane 9—p65, anti-p65 antibody. The relative intensities of EMSA results and the low-glucose control group were set as 1 (*n* = 4 in each group). (**C**) Immunofluorescence imaging of NF-kB and the staining of nuclear morphology by DAPI using fluorescence microscopy (*n* = 5 in each group). * *p* < 0.05, ** *p* < 0.01 compared with control cells incubated in low-glucose medium; # *p* < 0.05, ## *p* < 0.01 compared with ARPE-19 cells incubated in high-glucose medium. LG, low glucose; HG, high glucose; FGF-1, fibroblast growth factor type 1; GAPDH, glyceraldehyde-3-phosphate dehydrogenase.

To examine mitogen-activated protein kinase (MAPK) expression, we performed WB analysis to identify the activated phosphorylated form of MAPK. Phosphorylated p38 (Pp38) was activated in ARPE-19 cells incubated with a high-glucose medium for 24 h; however, no change was observed in p38 (Figure 6A). FGF-1 treatment inhibited Pp38 in an FGF-1 concentration–dependent manner.

To explore the effect of FGF-1 treatment on nuclear factor-kappa B (NF-κB) activity in ARPE-19 cells, we performed the electrophoretic mobility shift assay (EMSA) and IF analyses. In the EMSA experiment (Figure 6B), the nucleoprotein extracted from ARPE-19 cells in the high-glucose group exhibited increased NF-κB–DNA binding activity. The binding activity decreased after FGF-1 treatment. Consistent with the EMSA results, IF results revealed significantly increased NF-κB activation in ARPE-19 cells cultured in high-glucose medium and decreased NF-κB activation after FGF-1 treatment (Figure 6C). These results indicate that FGF-1 could prevent high-glucose-induced cell inflammation by suppressing the activation of p38 and NF-κB signaling.

## 3. Discussion

The pathogenesis of DR is multifaceted. Chronic loss of retinal neurons caused by increased oxidative stress and inflammatory angiogenic factors are the crucial mechanisms of diabetic neurodegeneration, which eventually leads to blindness [55,56]. Many studies have demonstrated that continuous hyperglycemia can cause oxidative stress and subsequent inflammation during DR development, and the loss of retinal neuronal cells cannot be reversed [56,57]. Therefore, treatments to prevent retinal neurodegeneration in patients with diabetes should be urgently developed. In the present study, our results demonstrated that FGF-1 treatment could alleviate diabetes-related retinal complications in a diabetic rat model in vivo and ARPE-19 cells in vitro. This effect was attributed to the reduction of oxidative damage and inflammation. One of the mechanisms might be related to the attenuation of p38 MAPK and NF-κB pathway activation by FGF-1 under high-glucose conditions.

The eyes are among several target organs involved in complications of diabetes [58]. The retina contains a large amount of polyunsaturated fatty acids in the outer segment of photoreceptor cells. In addition, the retina has a high glucose oxygen uptake reaction and is thus vulnerable to oxidative damage [59]. RPE cells are located between the neuroretina and choroidal vessels that act as the outer blood–retinal barrier. RPE cells play a key role in the metabolic support of retinal photoreceptors and are involved in the pathogenesis of DR [60]. In patients with diabetes mellitus, RPE cells are easily affected by hyperglycemia, which induces excessive ROS production [61]. ROS can destroy cell molecules and disturb normal cellular signal transduction, resulting in the apoptosis of RPE cells [62]. These findings indicate that RPE cells play a crucial role in diabetic eye-related conditions. The protection of RPE cells from high-glucose-induced damage is crucial in the treatment of DR. The use of ARPE-19 cells cultured in high-glucose medium as an in vitro model has been described in several studies on DR [63,64]. Therefore, ARPE-19 cells were used in the present study as an in vitro model, and the results demonstrated that FGF-1 administration reduced oxidative stress and cellular neuroinflammation in cells exposed to high-glucose medium.

FGF-1 is a member of a family of structurally related fibroblast growth factors. Initially, FGF-1 was isolated as a mitogen in the bovine brain and pituitary gland [27]. FGF-1 exerts autocrine and paracrine regulatory effects on cells. In addition, FGF-1 regulates mitosis in various tissues such as the liver, vascular system, and skin [28,29,30]. In addition, FGF-1 plays an important role in blood glucose regulation. Khuu et al. reported that the FGF-1 level in human AqH was significantly lower in patients with nonproliferative DR than in healthy controls [65]. Therefore, we hypothesized that the increased levels of FGF-1 molecular targets in the eyeballs can protect the retina from diabetic neurodegenerative damage. In the present study, we confirmed that FGF-1 expression in the retina of diabetic rats was downregulated, and intravitreous FGF-1 injection could reverse the insufficient FGF-1 level in the diabetic rat eyes.

Oxidative stress is considered to be the main cause of DR. This stress results in subsequent retinal cell death and fibrosis [66,67]. FGF-1 was found to prevent diabetic cardiomyopathy by reducing oxidative stress in mouse hearts [37]. Furthermore, FGF-1 treatment reduced diabetes-induced oxidative stress in mice with diabetic nephropathy [68,69]. Similarly, diabetes mellitus causes oxidative stress in the animal and human retina [70]. We investigated whether FGF-1 treatment ameliorated oxidative stress in DR. The expression levels of 8-OHdG, nitrotyrosine, and acrolein in the retinas of diabetic rats were assessed as a biomarker of oxidative stress caused by DNA, protein, and lipid peroxidation, respectively. In the present study, these oxidative damage biomarkers were markedly suppressed in diabetic rats treated with intravitreous injections of FGF-1. In addition, the expression levels of 8-OHdG, nitrotyrosine, and acrolein were significantly reduced by FGF-1 treatment in ARPE-19 cells cultured under high-glucose conditions. These results confirmed that FGF-1 treatment could block oxidative stress damage in the STZ-induced rat retina and ARPE-19 cells incubated in high-glucose medium, resulting in similar protective effects in both models.

Excessive oxidative stress activates transcription factors and the expression of proinflammatory genes. The development of neuronal inflammation is considered to be the main pathogenic mechanism of DR [56,71]. FGF-1 could effectively alleviate liver inflammation in a mouse model of nonalcoholic fatty liver disease [39]. In addition, FGF-1 reduced kidney inflammation in db/db mice [40,72] and ameliorated diabetic splenomegaly by suppressing inflammation (TNF-α, IL-1β, and IL-6) in db/db mice [73]. ICAM-1 is a crucial adhesion molecule that promotes the migration of white blood cells (leukostasis) and endothelial cells. MCP-1 is a cytokine that recruits monocytes, memory T cells, and dendritic cells to inflammation sites. IL-1β is a common proinflammatory cytokine, and IL-6 is a downstream inflammatory factor. These proteins can induce inflammation and tissue hypoxia and cause the migration of vascular endothelial cells, resulting in angiogenesis and worsened DR. Positive immunostaining was observed for ICAM-1 and MCP-1 in the retina of patients with diabetes [74]. Moreover, patients with DR exhibited significantly higher serum and AqH levels of proinflammatory cytokines IL-1β and IL-6 [75,76]. In the present study, the expression levels of the proinflammatory cytokines ICAM-1, MCP-1, IL-1β, and IL-6 were significantly higher in diabetic rats and high-glucose-treated ARPE-19 cells than in normal controls. FGF-1 treatment reduced the expression of proinflammatory products in the rat retina and ARPE-19 cells. These results indicated that diabetes upregulates the expression of neuroinflammation, which can be significantly suppressed by FGF-1 treatment.

MAPK signaling pathways are highly associated with mitogens and regulate many cellular processes. Extracellular signal-regulated kinase (ERK), c-Jun N-terminal kinase (JNK), and p38 kinase are MAPK families in mammalian cells. Among intracellular signaling systems in FGF-1, the MAPK signaling pathway is critical for regulating proinflammatory responses [77]. Liang et al. reported that FGF-1 can significantly inhibit kidney inflammation (i.e., cytokines and macrophage infiltration) and renal damage in diabetic nephropathy that is related to the NF-κB and c-Jun N-terminal (c-Jun) kinase signaling pathways [40]. Guillonneau et al. revealed that FGF-1 could activate ERK2 to reduce apoptosis in RPE cells [78]. Wang et al. determined that high-glucose-induced RPE barrier disruption could be prevented by suppressing p38 MAPK activation [79]. Furthermore, several studies have indicated that hyperglycemia could significantly enhance oxidative stress and proinflammatory cytokines in various types of cells through the ROS-mediated activation of p38 MAPK and the downstream transcription factor NF-κ signaling pathway [80,81,82,83]. NF-κB plays a key role in the pathogenesis of diabetic retinopathy [84]. Persistent hyperglycemia activates NF-κB, which increases the expression of various adhesion molecules and proinflammatory cytokines (i.e., ICAM-1, MCP-1, IL-1β, and IL-6) [19]. In the present study, we confirmed that a high-glucose condition enhanced the phosphorylation of p38 MAPK in a RPE cell model. FGF-1 treatment successfully inhibited p38 MAPK activation. Several studies have indicated that p38 MAPK activity adjusted the proinflammatory transcription factor NF-κB in various cell types [85,86,87,88]. In the present study, we observed that NF-κB p65 activation was significantly increased under high-glucose conditions and FGF-1 treatment inhibited NF-κB p65 activation. Taken together, our findings clarified that FGF-1 plays a regulatory role in the proinflammatory pathway by blocking the p38 MAPK pathway and inhibiting NF-κB p65 activation.

In conclusion, our findings indicate that FGF-1 could prevent the development of DR and the cascade of oxidative stress and neuroinflammatory signaling in the retinal tissues of STZ-induced diabetic rats and high-glucose-induced ARPE-19 cells by inhibiting the activation of p38 MAPK and NF-κB pathways. Therefore, FGF-1 treatment can be considered as a novel therapeutic target for preventing the development of diabetes-induced retinal neurodegeneration.

## 4. Materials and Methods

### 4.1. Animals

Male SD rats (age, 8 weeks; weight, approximately 270–300 g) were purchased from BioLASCO Taiwan Co. Ltd. The SD rats were placed in an environment under a 12-h light–dark cycle. The SD rats were divided into a diabetic experimental group (*n* = 10) and a normal control group (*n* = 5). Intraperitoneal injections of 55 mg/kg STZ (Sigma-Aldrich, St. Louis, MO, USA) were administered to induce diabetes in the rats. The SD rats in the control group were intraperitoneally injected with the same volume of citrate buffer. Blood glucose levels in the tail blood of the rats were measured 72 h after the intraperitoneal injection of STZ. Diabetes was considered to be successfully induced in the rats if the blood glucose level was >250 mg/dL.

### 4.2. Animal Experiments

FGF-1 (1.5 µg/2 µL; *n* = 10) or physiological saline (*n* = 5) was injected into the mid-vitreous of anesthetized rats by using a guide and needle technique at a dose of 2 µL every week for one month. Body weight and blood sugar levels were measured weekly. The SD rats were sacrificed through the intraperitoneal injection of a lethal dose of pentobarbital one week after the fourth injection. The eyeballs of each rat were immediately excavated and fixed with 4% paraformaldehyde in phosphate-buffered saline (PBS) for further pathological sectioning and IF staining. Several eyeballs were cut and centrifuged to obtain AqH, which was then used for ELISA.

### 4.3. Ethics Statement

This animal study complied with the Association for Research in Vision and Ophthalmology guidelines on the use of animals in ophthalmology and vision research. The protocol was approved by the Institutional Animal Care and Use Committee of the Institutional Animal Care and Use Committee of the National Taiwan University (permit number: 20180399, approval date: Dec 26, 2019).

### 4.4. Cell Culture and Treatment

Human ARPE-19 cells were purchased from the American Type Culture Collection (Manassas, VA, USA). The cells were maintained in a six-well plate and cultured in Dulbecco’s modified Eagle’s medium/F-12 human amniotic membrane nutrient mixture (DMEM/F-12; Invitrogen, Carlsbad, CA, USA) supplemented with 10% fetal bovine serum (Invitrogen) and penicillin/streptomycin solution (Invitrogen). The cells were incubated in a cell incubator at 37 °C in an atmosphere of 5% CO_2_. ARPE-19 cells were passaged to the next generation when they reached 80–90% confluence. Only cells within the third to fifth passages were used in the experiments.

In some cultures, a portion of the ARPE-19 cells were cultured in a low-glucose medium (5 mM; M131 medium, Invitrogen). The remaining cells were transferred to a high-glucose medium (25 mM; M131 medium (100 mL) + glucose solution (Gibco A2494001) (1.85 mL)). The cells were stimulated with different concentrations (0, 1, 5, or 10 ng/mL) of recombinant human FGF acidic (R&D Systems, Minneapolis, MN, USA; Catalog Number 232-FA) for 2 h before being transferred to a high-glucose medium (25 mM) for 24 or 72 h.

### 4.5. IF Detection of Oxidative Stress Markers and Inflammatory Mediators

The rat eyeball tissue sections and ARPE-19 cells were permeabilized in 0.2% Triton mixed with PBS containing 5% goat serum and incubated at 25 °C for 1 h. The sections and cells were then incubated with diluted primary antibodies in a blocking solution (Santa Cruz Biotechnology, Dallas, TX, USA) overnight at 4 °C. The following primary antibodies were used: anti-FGF1 antibody (ab179455), anti-8-OHdG (ab62623), antinitrotyrosine antibody (ab42789), antiacrolein antibody (ab37110), anti-ICAM-1 (10831-1-AP), anti-MCP-1 antibody (ab7202), and anti-IL-1β (12242S) antibody. VectaFluor horse antirabbit IgG, DyLight 594 antibody kit, R.T.U. (Catalog #: DI-1794), and DyLight 488 antibody kit, R.T.U. (Catalog #: DI-1788) were used as the secondary antibodies and incubated in a blocking solution at 25 °C for 3 h (Table 1). All steps were performed according to the manufacturer’s instructions. The nuclei were counterstained with 4′,6-diamidino-2-phenylindole (DAPI). Fluorescence intensities were measured using a microplate reader (Bio-Rad Laboratories, Hercules, CA, USA). The following formula was used for the densitometric quantitation of IF, as previously described [18] with a modification:

Immunostaining index = Σ [(X − threshold) × area (pixels)]/total cell number

where *X* is the staining density indicated by a number between 0 and 256 in grayscale, and *X* is above the threshold. In brief, digitized color images were obtained as PICT files. PICT files were opened in the grayscale mode by using ImageJ software (version 1.53a; NIH, Bethesda, MD, USA). Five images per experiment were analyzed, and the average was calculated. The relative density of immunostaining (fold change) was analyzed using an immunostaining index. The control (normal and nondiabetic) rats and ARPE-19 cells cultured in the low-glucose condition were set as normal reference, respectively.

### 4.6. Total RNA Extraction and Real-Time PCR Analysis

The TRIzol Reagent Kit (Invitrogen-Life Technologies, Carlsbad, CA, USA) was used to extract RNA, DNA, and protein from ARPE-19 cells for subsequent experimental analysis. A NucleoSPin RNA Kit (Macherey-Nagel GmbH & Co. KG, Düren, Germany) was used to extract the mRNA of all possible target genes, and the iScript cDNA Synthesis Kit (Bio-Rad) was used to reverse transcribe 2 µg of extracted mRNA into cDNA for subsequent real-time PCR (qPCR) experiments. An ABI StepOne real-time PCR system (Applied BioSystems, Foster City, CA, USA) and SYBR Green Master Mix reagent (Kapa Biosystems, Wilmington, MA, USA) were used. GAPDH was used as an endogenous control. All steps were performed according to the manufacturer’s instructions. The following primers were used for reactions (Table 2). qPCR results were analyzed using the 2^-ΔΔCt^ method, and the relative quantity of each mRNA was normalized to that of GAPDH mRNA. ABI StepOne Plus (Applied Biosystems) was used for data collection and analysis.

### 4.7. WB

Proteins were isolated from the rat retinal tissue and ARPE-19 cells by using lysis buffer containing 0.5 M Tris-HCl (pH 7.4), 10% NP-40, and a protein inhibitor (Roche Diagnostics, Rotkreuz, Switzerland). All lysates were centrifuged for 15 min at 12,000× *g* at 4 °C. The protein concentration was determined using the bicinchoninic acid (BCA) method (Pierce, Rockford, IL, USA). Forty micrograms of protein was subjected to electrophoresis in 10% sodium dodecyl sulfate–polyacrylamide gel electrophoresis. After electrophoresis, the separated proteins were transferred to the surface of polyvinylidene membranes (Immobilon-P; Millipore Corp., Billerica, MA, USA) by using TBS with 20% methanol. The membranes were incubated in PBS containing 5% milk at 25 °C for 1 h to block nonspecific bonds. Subsequently, the blots were incubated with the primary antibody (Table 3) and 5% milk at 4 °C overnight. The cells were washed with PBS containing Tween and incubated with secondary antibodies for 1 h at 25 °C (Table 3). An enhanced chemiluminescence detection system (Pierce Biotechnology, Waltham, MA, USA) was used to observe the results. The relative expression of proteins was measured through the densitometry analysis of blot bands by using ImageJ software. β-actin and GAPDH were used as internal controls.

### 4.8. ELISA

ELISA was used to measure ICAM-1, MCP-1, IL-1β, and IL-6 concentrations in rat AqH and ARPE-19 cell culture media. After the end of the experimental treatment, the supernatant was removed after centrifugation at 500× *g* for 10 min. Commercially available ELISA kits for ICAM-1 (cat. No. NBP1-92712; Novus Biologicals, Centennial, CO, USA), MCP-1 (Cat. No. 438807; BioLegend Inc., San Diego, CA, USA), IL-1β (Cat. No. EK0392; Boster Biological Technology, Pleasanton CA, USA), and IL-6 (Cat. No. 430508; BioLegend Inc., San Diego, CA, USA) were used. All steps were performed according to the manufacturer’s instructions. The specimen test solution was placed in a 96-well plate, and the optical density at 450 nm was measured at room temperature. The total protein content of each specimen was analyzed using the 20 Bradford assay (Bio-Rad Laboratories).

### 4.9. Nuclear Protein Extraction and NF-κB EMSA

The treated ARPE-19 cells were resuspended in 500 µL of buffer A solution (Bellco Glass, Vineland, NJ, USA). The samples were then homogenized through centrifugation at 1500× *g* at 4 °C for 10 min. The supernatant was removed, and the crude nuclear protein pellet was resuspended in 100 µL of buffer B solution. The sample was kept in an ice bath for 30 min and centrifuged at 15,000× *g* at 4 °C for 30 min. The supernatant containing the nuclear protein was transferred to a new tube and stored in an 80 °C freezer until use. The protein concentration was detected using the BCA method (Pierce) with 1 mg/mL of bovine serum albumin protein as the standard. A spectrophotometer was used to measure the absorbance of standard proteins at 595 nm.

EMSA was detected using a DNA binding NF-κB protein detection system (Pierce Biotechnology, Waltham, MA, USA). A 100-ng NF-κB oligonucleotide probe (5′-AGTTGAGGGGACTTTCCCAGGC-3′ and 3′-TCAACTCCCCTGAAAGGGTCC G-5′) was added to sterile water to a total volume of 10 µL. Thereafter, 4 µL of 5× labeling buffer, 4 µL of CoCl2-solution, 1 µL of DIG-ddUTP solution, and 1 µL of terminal transferase were mixed well at 37 °C for 15 min. A 2-µL volume of 0.2 M EDTA (pH 8.0) was placed on ice to complete the preparation of the probe. Nuclear protein (10 µg) and 5× buffer (50 mM Tris-HCl (pH 7.5), 500 mM NaCl, 5 mM dithreitol, 5 mM EDTA, 20% glycerol, and 2 µg of polydeoxyinosinic-deoxycytidylic acid) were reacted at 4 °C for 15 min. The specificity of DNA–protein binding was confirmed by adding a 100-fold molar excess of an unlabeled oligonucleotide for competitive binding 10 min before adding 3 µL of the DNA probe. The preparation was then left to react at room temperature for 20 min. The reaction was stopped through the addition of 1 mL of gel loading buffer, and electrophoresis was performed at 140 V by using 6% polyacrylamide gel and 0.5× TBE buffer. After electrophoresis, the film was transferred to a positively charged nylon membrane.

### 4.10. IF Staining of NF-κB

After we allowed ARPE-19 cells to grow in high-glucose medium (25 mM) for three days, the cells were fixed with cold methanol, blocked with 25% nonimmune goat serum (Sigma-Aldrich), incubated with the mouse antihuman NF-κB p65 antibody (1:100 diluted in PBS) covering the cell surface for 1 h at 37 °C, and then washed three times with PBS. Subsequently, fluorescein isothiocyanate-conjugated goat antimouse IgG (1:200 dilution) was applied for 1 h. The samples were washed three times with PBS (Jackson ImmunoResearch, West Grove, PA) and placed in a blocking solution at 25 °C for 3 h. All steps were performed according to the manufacturer’s instructions. Nuclei were counterstained with DAPI. The fluorescence intensity was measured using a fluorescent microplate reader (Bio-Rad Laboratories), and related images were captured.

### 4.11. Statistical Analyses

Data are presented as mean ± standard deviation. Statistical analyses of between-group differences were performed using Student’s t test and one-way analysis of variance, followed by Bonferroni’s test for multiple comparisons. Statistical significance was set at *p* < 0.05. All statistical analyses were performed using IBM SPSS for Windows (version 19.0; IBM, Armonk, NY, USA).

## Figures and Tables

**Table 1:** List of IF primary and secondary antibodies.

Name	Company	Catalogue Number	Concentration
**Primary Antibody**			
anti-FGF1	Abcam	ab179455	1:200
anti-8OHdG	Abcam	ab62623	1:200
anti-nitrotyrosine	Abcam	ab42789	1:200
anti-acrolein	Abcam	ab37110	1:200
anti-ICAM-1	Abcam	ab282575	1:200
anti-MCP-1	Abcam	ab7202	1:100
anti-IL-1β	Cell Signaling	12242S	1:200
**Secondary Antibody**			
DyLight 594 Antibody Kit	Vector	DI-1794	1:200
DyLight 488 Antibody Kit	Vector	DI-1788	1:200

**Table 2:** List of PCR primers.

Gene	Sequence (5′→3′)
ICAM-1	forward 5′-TCAGAAGGGACCGAGGTGAT-3′
	reverse 5′-TTTTCTGGCCACGTCCAGT-3′
MCP-1	forward 5′-CCAGATGCAATCAATGCCCC-3′
	reverse 5′-TCCTTGGCCACAATGGTCTT-3′
IL-1β	forward 5′-ACCTGAGCTCGCCAGTGAAA-3′
	reverse 5′-CAACAACTGACACGGCCTGC-3′
IL-6	forward 5′-AAGCCAGAGCTGTGCAAATGAG-3′
	reverse 5′-TCGTCAGCAGGCTGGCATTT-3′
GAPDH	forward 5′- TTCGAGAG-TCAGCCGCATTT-3′
	reverse 5′- GACTCCGACCTTCACCTTCC-3′

**Table 3:** List of WB primary and secondary antibodies.

Name	Company	Catalogue Number	Concentration
**Primary Antibody**			
anti-FGF1	Abcam	ab179455	1:1000
anti-ICAM-1	proteintech	10831-1-AP	1:500
anti-MCP-1	BioLegend	502601	1:1000
anti-IL-1β	Cell Signaling	12242S	1:1000
anti-IL-6	Abcam	ab6672	1:1000
anti-p38	Cell Signaling	#9212s	1:1000
anti-phospho-p38	Cell Signaling	#9211	1:1000
anti-GAPDH	Millipore	MAB374	1:2000
anti-β-actin	Abcam	ab8226	1:2000
**Secondary Antibody**			
anti-mouse IgG	Cell Signaling	7076S	1:5000
anti-rabbit IgG	Cell Signaling	7074S	1:5000

## Data Availability

Not applicable.
